# Impact of continuous hypertonic (NaCl 20%) saline solution on renal outcomes after traumatic brain injury (TBI): a post hoc analysis of the COBI trial

**DOI:** 10.1186/s13054-023-04311-1

**Published:** 2023-01-27

**Authors:** Olivier Huet, Xavier Chapalain, Véronique Vermeersch, Jean-Denis Moyer, Sigismond Lasocki, Benjamin Cohen, Claire Dahyot-Fizelier, Kevin Chalard, P. Seguin, Y. Hourmant, Karim Asehnoune, Antoine Roquilly, for the Atlanrea Study Group and the Société Française d’Anesthésie Réanimation (SFAR) Research Network

**Affiliations:** 1 Department of Anesthesiology and Surgical Intensive Care Unit, Brest University Hospital, Boulevard Tanguy Prigent, 29609 Brest, France; 2 Department of Anesthesiology and Critical Care, Beaujon Hospital, DMU Parabol, AP-HP Nord, Paris, France; 3 Department of Anesthesia and Intensive Care Unit, Angers Hospital, Angers, France; 4 Department of Anesthesia and Intensive Care Unit, Tours Hospital, Tours, France; 5 Department of Anesthesia and Intensive Care Unit, Poitiers Hospital, Poitiers, France; 6 Department of Anesthesia and Intensive Care Unit, Montpellier Hospital, Montpellier, France; 7 Department of Anesthesia and Intensive Care Unit, Rennes Hospital, Rennes, France; 8 Pôle Anesthésie Réanimations, Service d’Anesthésie Réanimation Chirurgicale, Hôtel Dieu, Université de Nantes, CHU Nantes, Nantes, France

**Keywords:** Acute kidney injury, Sodium chloride, Fluid therapy, Brain injury

## Abstract

**Background:**

To evaluate if the increase in chloride intake during a continuous infusion of 20% hypertonic saline solution (HSS) is associated with an increase in the incidence of acute kidney injury (AKI) compared to standard of care in traumatic brain injury patients.

**Methods:**

In this post hoc analysis of the COBI trial, 370 patients admitted for a moderate-to-severe TBI in the 9 participating ICUs were enrolled. The intervention consisted in a continuous infusion of HSS to maintain a blood sodium level between 150 and 155 mmol/L for at least 48 h. Patients enrolled in the control arm were treated as recommended by the latest Brain Trauma foundation guidelines. The primary outcome of this study was the occurrence of AKI within 28 days after enrollment. AKI was defined by stages 2 or 3 according to KDIGO criteria.

**Results:**

After exclusion of missing data, 322 patients were included in this post hoc analysis. The patients randomized in the intervention arm received a significantly higher amount of chloride during the first 4 days (intervention group: 97.3 ± 31.6 g vs. control group: 61.3 ± 38.1 g; *p* < 0.001) and had higher blood chloride levels at day 4 (117.9 ± 10.7 mmol/L vs. 111.6 ± 9 mmol/L, respectively, *p* < 0.001). The incidence of AKI was not statistically different between the intervention and the control group (24.5% vs. 28.9%, respectively; *p* = 0.45).

**Conclusions:**

Despite a significant increase in chloride intake, a continuous infusion of HSS was not associated with AKI in moderate-to-severe TBI patients. Our study does not confirm the potentially detrimental effect of chloride load on kidney function in ICU patients.

*Trial registration*: The COBI trial was registered on clinicaltrial.gov (Trial registration number: NCT03143751, date of registration: 8 May 2017).

**Supplementary Information:**

The online version contains supplementary material available at 10.1186/s13054-023-04311-1.

## Background

Traumatic brain injury (TBI) remains a worldwide health priority [1]. In 2016, 55 million patients suffered from TBI and it was responsible for 8.1 million years of life with disability [1]. At the initial phase of the insult, all medical and surgical interventions are focused on avoiding secondary cerebral insults [2]. In order to maintain an adequate cerebral perfusion pressure, intravenous fluid administration is one of the most common strategies prescribed by physicians [3, 4]. A recent consensus statement on fluid management for brain-injured patients suggested the use of crystalloids as the first-line choice for maintenance or resuscitation fluid [5]. The experts also stressed the poor level of proof to prefer buffered solution to normal saline solution (0.9% of NaCl) in brain-injured patients [5]. On the other hand, in case of refractory intracranial hypertension, hyperosmolar fluids (such as HSS) should be considered [5]. However, normal (0.9%) and hypertonic (3% or 20%) saline solutions are not devoid of side effects as saline solutions contain a “supra physiological” concentration of chloride that may induce metabolic disturbances [6]. Among them, hyperchloremia has been pointed out as an association has been reported between an increased concentration of extracellular chloride and an impairment of the vasotone of the afferent renal artery or a decrease in glomerular filtration rate [7–9]. These reports raised the question of the impact of hypertonic solutions on kidney function in neuro-intensive care patients.

Prospective and retrospective clinical studies have reported an association between chloride intake and AKI in critically ill patients [10–14]. However, these results remain controversial as a recent prospective multicenter study did not find any association between a “high dose” of chloride infusion and the renal prognosis in critically ill patients with septic shock [15, 16]. Moreover, the detrimental effect of saline solution on kidney function has not been consistently observed in the most recent randomized controlled trials [17–21]. Furthermore, the side effects of chloride-rich solution remain poorly studied in brain-injured patients, as it was underlined by a recent meta-analysis [22]. To our knowledge, only 3 small monocentric studies demonstrated metabolic disturbances induced by chloride-rich solution following brain injury, but they didn’t study the effect of HSS [23–25].

This post hoc analysis of the COBI trial aimed at determining whether high dose of chloride delivered by hypertonic saline solution infusion were associated with an increased acute kidney injury incidence within the 28 days after the infusion compared to standard care in TBI patients.

## Methods

### Design

This is a post hoc analysis of the COBI trial [26, 27]. Briefly, the COBI trial is a multicenter, randomized, open-label, controlled trial that has evaluated a continuous infusion of HSS (20% of NaCl) in a population of moderate-to-severe TBI [27]. Patients aged from 18 to 80 years old and admitted in one of the 9 participating ICU for moderate-to-severe TBI patients were eligible. Moderate-to-severe TBI was defined as the association of a Glasgow Coma Score (GCS) of 12 or lower and traumatic abnormal brain CT scan findings (such as: extradural hematoma, subdural hematoma, subarachnoid hemorrhage, brain contusion, brain hematoma, brain edema or skull fracture) [26]. In the interventional group, TBI patients received a 1-h bolus of 7.5 to 15 g of NaCl immediately after randomization. Then, a continuous infusion of HSS (1 g/h NaCl) was tapered to maintain a blood sodium level between 150 and 155 mmol/L for a minimum of 48 h. After 48 h, HSS (20%) was stopped in the absence of intracranial hypertension, and blood sodium level was maintained at a normal range (Na from 140 to 145 mmol/L). In case of a persistent intracranial hypertension, HSS infusion was maintained as long as necessary. In the control group, the latest Brain Trauma Foundation guidelines were applied as the standard of care [28]. The current post hoc analysis uses patient-level data focused on metabolic disturbances and kidney function to evaluate potential side effects associated with a continuous infusion of HSS (20%).

### Ethical approval

The current study was approved by the Ethics Committee of Ile de France VIII in May 2017 and conducted in accordance with the principles of the Declaration of Helsinki. Written informed consent was provided to all eligible patients at inclusion or after they recovered the ability to consent. In the other cases, the next of kin provided informed consent. Consent to participate included a statement that the current study was carried out in accordance with the principles of the Declaration of Helsinki.

### Data collection

We collected some physiological measurements to describe metabolic status. From the inclusion to day 4, the following parameters were collected daily: chloride, potassium, pH, bicarbonate, lactate, urea and creatinine. Blood sodium levels were collected from inclusion to Day 10. For these metabolic parameters, the worst value each day was collected. The highest weekly creatinine levels were collected from inclusion to Day 28. We calculated Glomerular Filtration Rate (GFR) according to the Modification of Diet in Renal Disease (MDRD)-175 equation. Estimated Glomerular Filtration Rate (eGFR) was calculated daily from inclusion to Day 4, and the worst eGFR each week from Week 1 to Week 4. The proportion of patients exposed to hyperchloremia (chloride level ≥ 110 mmol/L) was also reported. We defined metabolic acidosis using the following parameters: pH < 7.35 and bicarbonate level < 22 mmol/L. Urine outputs were collected from inclusion to Day 4. We also collected the amount of chloride received daily (g/day) during the first 4 days.

### Primary outcome

The primary outcome was the proportion of patients developing an AKI from inclusion to Day 28. Kidney function was evaluated according to the KDIGO criteria such as: Serum creatinine (µmol/L) and Urine output (mL/kg/h) [29]. AKI was defined as a KDIGO stage 2 or 3 [29]. All data were analyzed, even those from patients who died before day 28. No censure was applied in the analysis.

### Secondary outcomes

First, we planned to evaluate the association between the use of the continuous infusion of hypertonic saline solution and the occurrence of hyperchloremia and metabolic acidosis. Second, we studied the association between the continuous infusion of HSS and the need for renal replacement therapy (RRT) within 28 days after starting the infusion of HSS. Third, we studied the association of AKI from inclusion to Day 28 with chloride level and cumulative chloride dose infused. Finally, we tested the association between AKI and ICU length of stay, the need of RRT from inclusion to Day 28, and ICU mortality. As an exploratory analysis, we also compared eGFR and the highest creatinine levels between the two groups.

### Statistical analysis

Continuous variables were expressed as mean (± SD) or median (IQR). Categorical variables were expressed as percentage. Missing data were identified, and multiple imputations (5 iterations) were used for variables with less than 20% missing data. Continuous variables normally distributed were compared with unpaired Student t-tests. A Wilcoxon test was used for other continuous variables. Categorical variables were compared with the Chi-Square test. In univariate analysis, we identified the unbalanced variables between the two groups (control vs. interventional arm) and any variables associated with AKI from inclusion to Day 28. For repeated measures comparison, we performed a mixed model with a “subject” random effect variable and a “time” fixed effect variable. Results of the mixed model are shown as coefficients and estimated *p* value according to Satterthwaite approximation method. Statistical significance was set at *p* < 0.05. All statistical analysis was performed using R statistical software (version 3.6).

## Results

### Patients

A total of 370 patients underwent randomization in the COBI study. Forty-eight (13%) patients were excluded from this post hoc analysis for missing data for the primary outcome. Compared to excluded patients (*n* = 48), patients included in this post hoc analysis were more frequently exposed to hypotension, hypoxemia or a drop of hemoglobin level < 9 g/dL before inclusion (Additional file 2: Table S2). Included patients had also more frequently a past medical history of chronic kidney disease. These results are summarized in Additional file 2: Table S2. Of the 322 remaining patients, 159 (49.4%) patients were randomized in the control group and 163 (50.6%) patients in the interventional group. The main patients’ characteristics at baseline are described in Table 1. At baseline, the chloride level was 106.5 in the control group versus 107.3 mmol/L in the intervention group (*p* = 0.18). The proportions of patients with chronic kidney disease (2.5% vs. 2.5%, respectively; *p* = 1.00) and baseline eGFR (109.6 vs. 111.5 mL/min/1.73 m^2^; *p* = 0.65) were also balanced between the two groups.

**Table 1:** Baseline characteristics in the two groups

	Control	Intervention	*p*
	*n* = 159	*n* = 163	
Age, years mean (SD)	44.1 (17.6)	43.2 (17.7)	0.6
Sex, male *n* (%)	129 (81.1)	127 (77.9)	0.2
Weight, kg mean (SD)	79 (17.7)	73.6 (15.3)	0.02
Time from trauma to inclusion, Hours mean (SD)	12.7 (6.2)	13 (6.4)	0.67
Severe TBI, *n* (%)	139 (87.4)	142 (87.1)	1
ICP before inclusion, mmHg mean (SD)	12.9 (9.3)	10.8 (7.1)	0.051
MAP before inclusion, mmHg mean (SD)	86.3 (15.3)	85.2 (14.9)	0.51
Hypotension, *n* (%)	26 (16.4)	26 (16)	1
Hypoxemia, *n* (%)	25 (15.7)	26 (16)	1
Hemoglobin level < 9 g/dl, *n* (%)	14 (8.8)	12 (7.4)	0.79
Chronic kidney disease, *n* (%)	4 (2.5)	4 (2.5)	1
eGFR, mL/min/1.73 m^2^	109.6 (39)	111.5 (34.8)	0.65
Hyperosmolar therapy, *n* (%)	94 (59.1)	91 (55.8)	0.63
Mannitol	52 (55.3)	57 (62.6)	0.39
Hypertonic saline solution	63 (67.0)	57 (62.6)	0.64
*Baseline metabolic parameters, mean (SD)*			
Sodium, mmol/L	140 (3.9)	140.4 (4.6)	0.42
Chloride, mmol/L	106.5 (5.2)	107.3 (5.4)	0.18
Urea, mmol/L	4.8 (1.9)	4.9 (3.2)	0.87
Creatinine, µmol/L	73.6 (30.4)	72.4 (31.9)	0.74
Acid base status			
Arterial pH	7.34 (0.09)	7.34 (0.09)	0.97
Base deficit, mmol/L	− 3.73 (3.69)	− 3.49 (3.70)	0.56
Bicarbonate, mmol/L	21.67 (3.42)	22.04 (2.78)	0.28
Arterial CO_2_, mmHg	41.24 (8.07)	42.96 (17.48)	0.26
Lactate level, mmol/L	2.40 (2.31)	2.25 (1.74)	0.51
Lactate > 2 mmol/L, *n* (%)	73 (45.9)	62 (38.0)	0.19
*Daily chloride load, g/day mean (SD)*			
Before inclusion	14.9 (11.2)	22.7 (25.8)	0.001
Day 1	17.5 (14.8)	34.3 (13.9)	< 0.001
Day 2	12 (7.3)	19.7 (9.7)	< 0.001
Day 3	8.9 (6.7)	11.2 (8.7)	0.008
Day 4	8 (7)	9.4 (7.7)	0.09
Cumulative chloride load at Day 4, g mean (SD)	61.3 (31.6)	97.3 (38.1)	< 0.001
Antidiuretic hormone use during ICU stay, *n* (%)	26 (16.4)	10 (6.1)	0.006
*Outcomes*			
ICU length of stay, Days mean (SD)	20.4 (17.9)	23.7 (22.1)	0.14
ICU mortality, *n* (%)	32 (20.1)	26 (16)	0.41

*eGFR* estimated Glomerular Filtration Rate, *ICP* Intracranial Pressure, *ICU* Intensive Care Unit, *MAP* Mean Arterial Pressure, *SD* Standard Deviation, *TBI*: Trauma Brain Injury

### Chloride intake and metabolic parameters

**Fig. 1:**
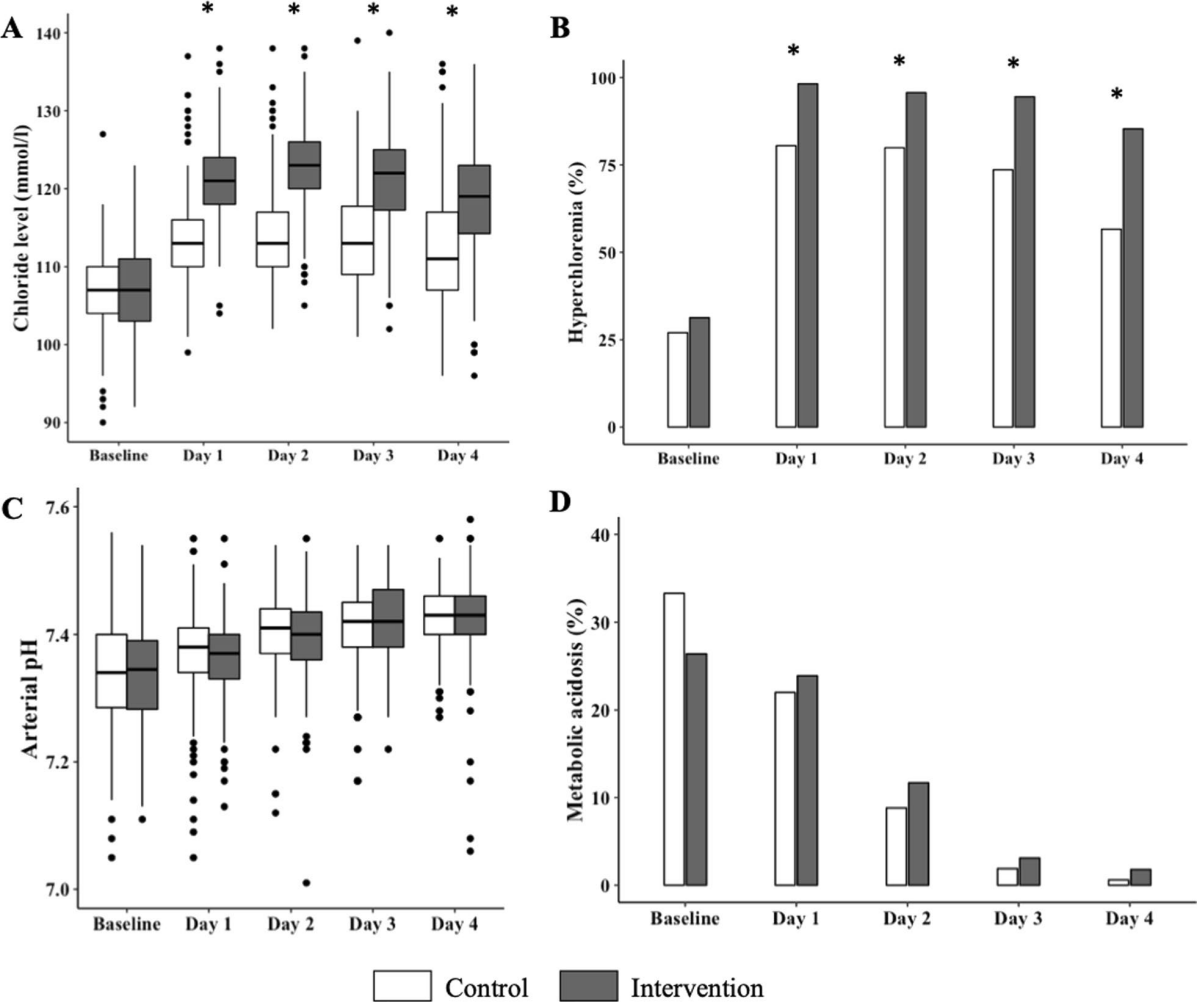
Trend in main metabolic parameters measured from inclusion to Day 4 in the two groups. **A** Higher chloride level (mmol/L) from inclusion to Day 4. **B** Proportion of patients exposed to hyperchloremia (Cl > 109 mmol/L) at each time from inclusion to Day 4. **C** Lower pH level from inclusion to Day 4. **D** Proportion of patients exposed to metabolic acidosis (pH < 7.35 and bicarbonate level < 22 mmol/L) at each time from inclusion to Day 4. In each boxplot, dots represented outliers. *p < 0.05 for univariate analysis

Patients randomized in the interventional arm received a continuous infusion of hypertonic saline solution for a mean of 2.6 (± 1.3) days. The cumulative chloride load received from ICU admission to Day 4 was 97.3 ± 38.1 g in the intervention group and 61.3 ± 31.6 g in the control group (*p* < 0.001). The time course of the recorded biological parameters is summarized in Table 2. From Day 1 to Day 4, the daily chloride levels were significantly higher for patients randomized in the interventional group (*p* < 0.001). This univariate analysis was confirmed by a mixed model which found a higher slope in chloride trend in the intervention arm from Day 1 to Day 4 with a regression coefficient of 5.8 (*p* < 0.001). From day 1 to day 4, the base deficit, arterial pH, and bicarbonate levels were not statistically different between the two groups. Repeated measures were analyzed using a mixed model. We did not find any between-group differences in terms of base deficit, arterial pH and bicarbonate level. These results are summarized in Table 2. The time course of chloride levels and arterial pH in the two study groups are shown in Fig. 1 with the results of the mixed model. The occurrence of metabolic acidosis from Day 1 to Day 4 was not statistically different between the two groups (Fig. 1).

**Table 2:** Comparison of metabolic parameters between the two groups

	Control	Intervention	*p*
	*n* = 159	*n* = 163	
*Sodium, mmol/L*			
Before inclusion	140 (3.9)	140.4 (4.6)	0.42
Day 1	145 (5.7)	151.9 (4.9)	< 0.001
Day 2	145.6 (6.3)	154.1 (5.4)	< 0.001
Day 3	146.3 (6.5)	153.8 (5.8)	< 0.001
Day 4	145.9 (6.4)	151.7 (6.2)	< 0.001
*Chloride, mmol/L*			
Before inclusion	106.5 (5.2)	107.3 (5.4)	0.18
Day 1	113.6 (6)	120.8 (8.1)	< 0.001
Day 2	114.5 (6.4)	122.8 (8.6)	< 0.001
Day 3	114.6 (8.8)	121 (8.5)	< 0.001
Day 4	111.6 (10.7)	117.9 (9)	< 0.001
*Arterial pH*			
Before inclusion	7.34 (0.09)	7.34 (0.09)	0.97
Day 1	7.37 (0.08)	7.36 (0.06)	0.46
Day 2	7.40 (0.07)	7.39 (0.07)	0.22
Day 3	7.41 (0.07)	7.42 (0.06)	0.24
Day 4	7.43 (0.05)	7.42 (0.07)	0.58
*Base deficit, mmol/L*			
Before inclusion	− 3.7 (3.7)	− 3.5 (3.7)	0.56
Day 1	− 2.7 (3.6)	− 2.7 (3.1)	0.97
Day 2	− 0.47 (2.9)	− 0.96 (2.9)	0.13
Day 3	1.2 (2.7)	0.9 (2.5)	0.28
Day 4	2.3 (3.3)	2.4 (3)	0.98
*Bicarbonate, mmol/L*			
Before inclusion	21.7 (3.4)	22.1 (2.8)	0.28
Day 1	16.4 (10.8)	17 (10.1)	0.62
Day 2	23.5 (3.2)	23.1 (2.6)	0.25
Day 3	25.1 (3.1)	24.9 (2.3)	0.59
Day 4	27 (5)	27.2 (4.3)	0.70
*Lactate, mmol/L*			
Before inclusion	2.4 (2.3)	2.3 (1.7)	0.51
Day 1	1.8 (1.2)	1.6 (0.8)	0.21
Day 2	1.4 (0.8)	1.5 (1)	0.75
Day 3	1.3 (0.5)	1.3 (0.6)	0.58
Day 4	1.2 (0.49)	1.2 (1)	0.90

Results were expressed as means (± SD)

*SD* Standard Deviation

### Primary outcome

From inclusion to Day 28, AKI was recorded in 46 (28.9%) patients in the control group and 40 (24.5%) in the intervention group (*p* = 0.45, Table 3). The great majority of AKI event (96.5%) was observed before Day 7 without differences between the two groups: 46 (28.9%) in control group vs. 37 (22.7%) in intervention group (*p* = 0.25). Only 3 patients suffered from AKI after day 7, these patients were in the intervention arm.

**Table 3:** Comparison of renal outcomes in the 2 groups from inclusion to Day 28

	Control	Intervention	*p*
	*n* = 159	*n* = 163	
*From inclusion to Day 28*			
AKI (KDIGO 2–3), *n* (%)	46 (28.9)	40 (24.5)	0.45
KDIGO classification, *n* (%)			0.62
1	32 (20.1)	30 (18.4)	
2	35 (22)	33 (20.2)	
3	11 (6.9)	7 (4.3)	
RRT, *n* (%)	3 (1.9)	3 (1.8)	1

*AKI* Acute Kidney Injury, *KDIGO* Kidney Disease Improving Global Outcome, *RRT* Renal Replacement Therapy, *SD* Standard Deviation

### Renal outcomes

**Fig. 2:**
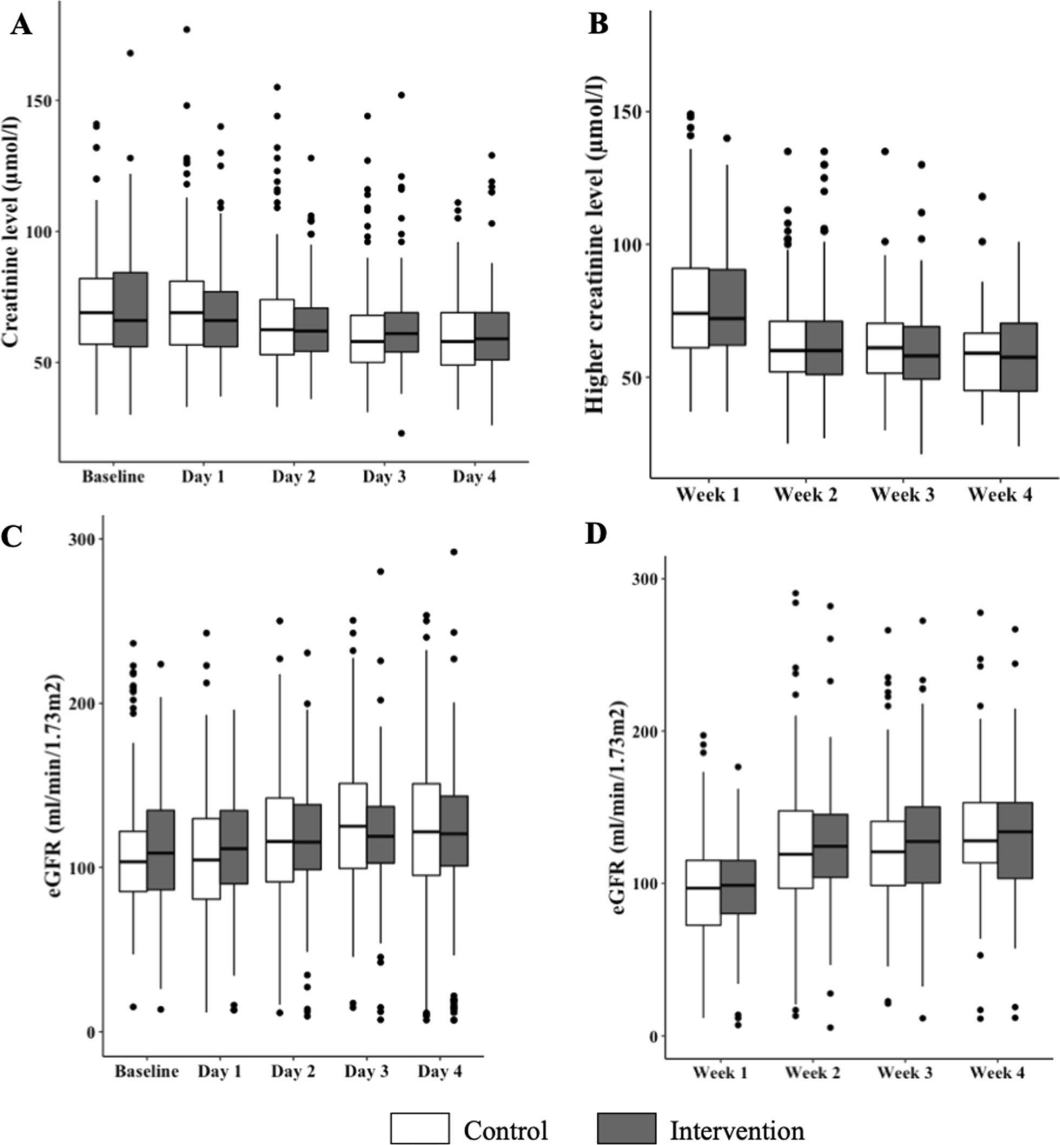
Trend in kidney parameters measured in the two groups. **A** Creatinine level (µmol/L) measured from inclusion to Day 4. **B** Higher creatinine level (µmol/L) measured each week from Week 1 to Week 4. **C** Daily eGFR (mL/min/1.73 m^2^) from inclusion to Day 4. **D** Worst eGFR (mL/min/1.73 m^2^) measured each week from Week 1 to Week 4. In each boxplot, dots represented outliers. *eGFR* estimated Glomerular Filtration Rate

During the ICU stay, 3 (1.9%) patients in the control group and 3 (1.8%) in the intervention group received RRT (*p* = 1). The main reason for beginning RRT was: severe metabolic acidosis (3 patients), severe hyperkalemia (1 patient), elevated blood urea nitrogen (1 patient), and pulmonary edema (1 patient). The time course of the creatinine levels and eGFR during the first four days were not statistically different between the two groups from inclusion to Day 4. This was also the case for the highest weekly creatinine levels and lowest eGFR from inclusion to Day 28 (Fig. 2). The mixed model analysis confirmed the univariate analysis. In this random effect model, trends of creatinine level and eGFR were also comparable (*p* value > 0.05).

### Association of AKI with TBI patients’ outcomes

As an exploratory analysis, we studied the association between AKI onset, defined as a KDIGO stage 2 and 3, and several ICU outcomes. The length of ICU stay was 19.8 ± 17.4 days in TBI patients without AKI and 28.2 ± 25.4 days in patients with AKI (p = 0.001). ICU mortality was 19.5% for patients without AKI and 14% for patients with AKI (p = 0.33). There was no association between cumulative chloride infusion on Day 4 and the occurrence of AKI: 79.2 g versus 80.4 g (*p* = 0.81). There was no association between hyperchloremia exposure from inclusion to Day 4 and the onset of AKI within 28 days after randomization. These results are summarized in Additional file 1: Table S1.

## Discussion

This post hoc analysis of the COBI trial evaluated the impact of a continuous infusion of HSS (20%) on renal function after moderate-to-severe TBI. No association between the continuous infusion of HSS (20%) and AKI (KDIGO stage 2 or 3) from inclusion to Day 28 was find. Creatinine levels and eGFR were also similar between the two groups. Chloremia was significantly higher in the HSS group. No difference in acid/base metabolic status was found between the two groups.

Fluid therapy solutions, administered in ICU, contain different concentrations of electrolytes [3]. Among these electrolytes, chloride has raised concern about its iniquity [3]. Actually, an acute administration of a large amount of chloride-rich solution may lead to hyperchloremic metabolic acidosis associated with pathophysiological consequences such as: coagulopathy, hypotension, or splanchnic disorders [30, 31]. Furthermore, chronic hyperchloremia is also theoretically associated with an excessive vasoconstriction of afferent renal arteries leading to a decrease in glomerular filtration rate [7–9].

In the past decade, three large RCTs compared buffered crystalloid to normal (0.9%) saline solution in various settings [17, 19–21]. These RCT’s did show a statistically significant difference between the groups regarding chloride intake or changes in chloremia and arterial acid–base balance [17, 19–21]. However, the clinical significance of these differences is questionable. Actually, in the SMART trial the difference of chloride intake between the two groups was of 0.5 g, and differences in terms of blood chloride levels were also small and probably not clinically relevant: 109 mmol/L (saline solution) versus 108 mmol/L (balanced crystalloid) [20]. These limitations are also found in the BaSICS and the PLUS trials [17, 19, 21]. Therefore, it is difficult to conclude about the kidney toxicity of high chloride intake in ICU patients with these clinical trials [32].

On the other hand, patients enrolled in the COBI Trial received a substantial and prolonged amount of chloride in the interventional arm. To our knowledge, this is the first time that the effect of such an important chloride intake has been reported. Our results are not in line with a potential toxicity of chloride intake on kidney function. To date, there was only one large RCT comparing HSS (3%) to normal saline solution (0.9%) and the proportion of patients who required RRT [33]. In this study, the needed of RRT between HSS and normal saline solution was not statistically different (38% vs. 33%, *p* = 0.32) [33].

The main strength of our study is the design, as it is a randomized controlled trial. Second, this is the first study reporting the effect of the infusion of a highly concentrated solution in chloride. Third, we report the amount of chloride infused and the difference in blood chloride levels which is rarely done in other studies.

Our study also has some limitations. First, this is a post hoc analysis, and this ancillary study was not planned in the original statistical analysis plan [27]. Second, patients in the control group also received a significant amount of chloride (61.3 ± 31.6 g in 4 days) and were also exposed to hyperchloremia from Day 1 to Day 4. This may have limited the statistical power of this study to demonstrate a difference between the two study groups. However, the amount of chloride infused in the control group is comparable to the estimated amount reported in the recent CENTER-TBI cohort [34]. Moreover, in our study the chloride intake is clinically significantly higher in the intervention arm. Third, patients included in the COBI trial were younger (mean age: 44 years old) and had a lower risk of AKI than patients included in previous studies [18, 20]. Moreover, the requirement of RRT (1.9%) was slightly lower compare to other studies [17, 20]. Thus, our results cannot be generalized to high risk patients. Fourth, AKI patients had more frequently a baseline chronic kidney disease but the proportion of patients who had a chronic kidney disease was well balanced between control and intervention group. Therefore, we believe that the analysis of the main results cannot be confounded by chronic kidney disease.

## Conclusion

After moderate-to-severe TBI, the continuous infusion of HSS (20%) was not associated with an increased the risk of developing AKI from inclusion to Day 28. Our findings questioned the potentially detrimental effects of chloride-rich solution on kidney function. Further studies are warranted to better evaluate impact of fluid therapy in ICU patients, especially after TBI.

### Supplementary Information

**Additional file 1: Table S1**. Comparison between patients according to existence of AKI (KDIGO stage 2–3) from inclusion to Day 28.

**Additional file 2: Table S2**. Comparison of baseline characteristics between included and non-included patients.

## Data Availability

The dataset supporting the conclusions of this article is fully available. To have an access on it, please contact the corresponding author (O.H.)

## Abbreviations

AKI: Acute Kidney Injury
eGFR: Estimated Glomerular Filtration Rate
GCS: Glasgow Coma Scale
GFR: Glomerular Filtration Rate
ICP: Intracranial Pressure
ICU: Intensive Care Unit
IQR: Inter Quartile Range
KDIGO: Kidney Disease Improving Global Outcome
MAP: Mean Arterial Pressure
MDRD: Modification of Diet in Renal Disease
RCT: Randomized Controlled Trial
RRT: Renal Replacement Therapy
SD: Standard Deviation
TBI: Trauma Brain Injury
