# Supplementary material for: Impact of continuous hypertonic (NaCl 20%) saline solution on renal outcomes after traumatic brain injury (TBI): a post hoc analysis of the COBI trial
Source: Crit Care. 2023 Jan 27;27:42. doi: 10.1186/s13054-023-04311-1 (PMC9881296; doi:10.1186/s13054-023-04311-1)
Supplement: Supplementary file 1 — Additional file 1: Table S1. Comparison between patients according to existence of AKI (KDIGO stage 2–3) from inclusion to Day 28. [file 13054_2023_4311_MOESM1_ESM.docx]

**Table S1:** Comparison between patients according to existence of AKI (KDIGO stage 2-3) from inclusion to Day 28.

|  | **AKI from inclusion to Day 28** | |  |
| --- | --- | --- | --- |
|  | **No** | **Yes** | **P** |
|  | **n = 236** | **n = 86** |  |
| Age, years mean (SD) | 42.8 (17.6) | 46 (17.5) | 0.16 |
| Sexe, Male n (%) | 185 (78.4) | 71 (82.6) | 0.47 |
| Severe TBI, n (%) | 207 (87.7) | 74 (86) | 0.84 |
| Hyperosmolar therapy, n (%) | 133 (56.4) | 52 (60.5) | 0.59 |
| Mannitol | 79 (59.4) | 30 (57.7) | 0.96 |
| Hypertonic saline solution | 84 (63.2) | 36 (69.2) | 0.54 |
| Hypotension, n (%) | 40 (16.9) | 12 (14) | 0.64 |
| Hypoxemia, n (%) | 35 (14.8) | 16 (18.6) | 0.52 |
| Chronic kidney disease, n (%) | 3 (1.3) | 5 (5.8) | 0.03 |
| MAP before inclusion, mean (SD) | 84.9 (14.8) | 87.9 (15.8) | 0.12 |
| **Proportion of patients in interventional arm, n (%)** | 123 (52.1) | 40 (46.5) | 0.45 |
| **Chloride infusion, g/day mean (SD)** |  |  |  |
| Before inclusion | 18.6 (22.3) | 19.5 (13.7) | 0.73 |
| Day 1 | 26.2 (17.5) | 25.4 (13.9) | 0.71 |
| Day 2 | 16.1 (10) | 15.3 (7.5) | 0.49 |
| Day 3 | 10 (8) | 10.3 (7.4) | 0.78 |
| Day 4 | 8.2 (7.2) | 9.9 (7.9) | 0.08 |
| **Cumulative chloride infusion, g mean (SD)** | 79.2 (41.3) | 80.4 (33.8) | 0.81 |
| **Biological parameters** |  |  |  |
| Hyperchloremia, n (%) |  |  |  |
| Before inclusion | 73 (30.9) | 21 (24.4) | 0.32 |
| Day 1 | 213 (90.3) | 75 (87.2) | 0.56 |
| Day 2 | 208 (88.1) | 75 (87.2) | 0.97 |
| Day 3 | 198 (83.9) | 73 (84.9) | 0.97 |
| Day 4 | 162 (68.6) | 67 (77.9) | 0.14 |
| Chloride, mean (SD) |  |  |  |
| Before inclusion | 107.2 (5.5) | 106.1 (4.9) | 0.1 |
| Day 1 | 117.5 (8.4) | 116.6 (6.8) | 0.4 |
| Day 2 | 118.6 (9) | 118.9 (7.5) | 0.85 |
| Day 3 | 117.3 (8.6) | 119.2 (10.5) | 0.09 |
| Day 4 | 114.4 (10.7) | 116 (9.46) | 0.23 |
| **Outcomes** |  |  |  |
| ICU length of stay, Days mean (SD) | 19.8 (17.4) | 28.2 (25.4) | 0.001 |
| RRT during ICU stay, n (%) | 2 (0.8) | 4 (4.7) | 0.046 |
| ICU mortality, n (%) | 46 (19.5) | 12 (14) | 0.33 |

AKI: Acute Kidney Injury ; ICP: Intracranial Pressure ; ICU: Intensive Care Unit ; KDIGO: Kidney Disease Improving Global Outcome ; MAP: Mean Arterial Pressure ; TBI: Trauma Brain Injury ; RRT: Renal Replacement Therapy ; SD: Standard Deviation
