# Supplementary material for: Impact of continuous hypertonic (NaCl 20%) saline solution on renal outcomes after traumatic brain injury (TBI): a post hoc analysis of the COBI trial
Source: Crit Care. 2023 Jan 27;27:42. doi: 10.1186/s13054-023-04311-1 (PMC9881296; doi:10.1186/s13054-023-04311-1)
Supplement: Supplementary file 2 — Additional file 2: Table S2. Comparison of baseline characteristics between included and non-included patients. [file 13054_2023_4311_MOESM2_ESM.docx]

|  | **Patients included** | | **P** |
| --- | --- | --- | --- |
|  | **No** | **Yes** |  |
|  | **n = 48** | **n = 322** |  |
| Age, years mean (SD) | 42.5 (17.8) | 43.9 (17.6) | 0.61 |
| Sexe, Male n (%) | 36 (75) | 256 (79.5) | 0.19 |
| Time from trauma to inclusion, Hours mean (SD) | 12.6 (6.4) | 13 (6.3) | 0.7 |
| GCS, mean (SD) | 7 (2.8) | 6.7 (2.8) | 0.55 |
| ICP before inclusion, mmHg mean (SD) | 13.1 (9.2) | 11.6 (8.2) | 0.27 |
| MAP before inclusion, mmHg mean (SD) | 83.9 (12.8) | 85.75 (15) | 0.43 |
| Hypotension, n (%) |  |  | 0.001 |
| Missing data | 3 (6.2) | 1 (0.3) |  |
| No | 39 (81.2) | 270 (83.9) |  |
| Yes | 6 (12.5) | 51 (15.8) |  |
| Hypoxemia, n (%) |  |  | <0.001 |
| Missing data | 3 (6.2) | 1 (0.3) |  |
| No | 41 (85.4) | 270 (83.9) |  |
| Yes | 4 (8.3) | 51 (15.8) |  |
| Hemoglobin level < 9 g/dl, n (%) |  |  | 0.001 |
| Missing data | 3 (6.2) | 1 (0.3) |  |
| No | 42 (87.5) | 295 (91.6) |  |
| Yes | 3 (6.2) | 26 (8.1) |  |
| Chronic kidney disease, n (%) |  |  | 0.001 |
| Missing data | 3 (6.2) | 1 (0.3) |  |
| No | 45 (93.8) | 313 (97.2) |  |
| Yes | 0 (0) | 8 (2.5) |  |
| Neurosurgery before inclusion, n (%) |  |  | 0.02 |
| Missing data | 3 (6.2) | 3 (0.9) |  |
| No | 35 (72.9) | 231 (71.7) |  |
| Yes | 10 (20.8) | 88 (27.3) |  |

**Table S2:** Comparison of baseline characteristics between included and non-included patients.

GCS: Glasgow Coma Scale ; ICP: Intracranial Pressure ; MAP: Mean Arterial Pressure ; SD: Standard Deviation
